# Plant Antiviral Immunity Against Geminiviruses and Viral Counter-Defense for Survival

**DOI:** 10.3389/fmicb.2019.01460

**Published:** 2019-06-26

**Authors:** R. Vinoth Kumar

**Affiliations:** National Centre for Biological Sciences, Tata Institute of Fundamental Research, Bengaluru, India

**Keywords:** virus, macromolecular trafficking, post-translational modification, hormone signaling, antiviral response, vector transmission, viral pathogenesis

## Abstract

The family *Geminiviridae* includes plant-infecting viruses whose genomes are composed of one or two circular non-enveloped ssDNAs(+) of about 2.5–5.2 kb each in size. These insect-transmissible geminiviruses cause significant crop losses across continents and pose a serious threat to food security. Under the control of promoters generally located within the intergenic region, their genomes encode five to eight ORFs from overlapping viral transcripts. Most proteins encoded by geminiviruses perform multiple functions, such as suppressing defense responses, hijacking ubiquitin-proteasomal pathways, altering hormonal responses, manipulating cell cycle regulation, and exploiting protein-signaling cascades. Geminiviruses establish complex but coordinated interactions with several host elements to spread and facilitate successful infection cycles. Consequently, plants have evolved several multilayered defense strategies against geminivirus infection and distribution. Recent studies on the evasion of host-mediated resistance factors by various geminivirus proteins through novel mechanisms have provided new insights into the development of antiviral strategies against geminiviruses. This review summarizes the current knowledge concerning virus movement within and between cells, as well as the recent advances in our understanding of the biological roles of virus-encoded proteins in manipulating host-mediated responses and insect transmission. This review also highlights unexplored areas that may increase our understanding of the biology of geminiviruses and how to combat these important plant pathogens.

## Introduction

**FIGURE 1:**
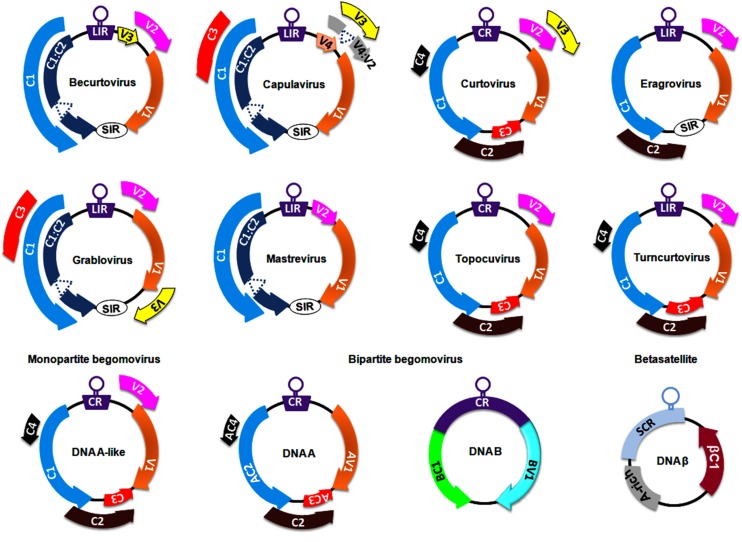
Genomeorganization of viruses belonging to different genera of the family, *Geminiviridae*. The unfilled circles indicate the origin of replication, and all the coding regions are labeled according to the function of their respective gene products viz., C1/AC1: replication associated protein (Rep); C2/AC2: transcriptional activator protein (TrAP); C3/AC3: replication enhancer protein (REn); V1/AV1: coat protein (CP); V2/AV2: pre-coat protein; BC1: movement protein (MP) and BV1: nuclear shuttle protein (NSP). CR, SCR, SIR, and LIR refer to Common Region, Satellite Conserved Region, Short-Intergenic Region, and Long-Intergenic Region, respectively.

The *Geminiviridae* family includes a group of circular ssDNA viruses that cause severe diseases in numerous crops (including monocots and dicots) and weeds, resulting in significant crop losses worldwide (Navas-Castillo et al., 2011). At the genus level, the *International Committee on Taxonomy of Viruses* has classified the *Geminiviridae* family into nine genera, *Becurtovirus, Begomovirus, Capulavirus, Curtovirus, Eragrovirus, Grablovirus, Mastrevirus, Topocuvirus*, and *Turncurtovirus*, based on insect vector, genome organization, host range, and genome-wide pairwise sequence identities (Zerbini et al., 2017) (Figure 1). Several factors such as recombination, pseudo-recombination, microsatellites, mutations, nucleotide substitutions, synergism, and vector-mediated transmission drive the rapid emergence and evolution of geminiviruses (Rojas et al., 2005; Lefeuvre and Moriones, 2015; Czosnek et al., 2017; Kumar and Chakraborty, 2018).

Members of the *Mastrevirus* and *Begomovirus* genera are widely studied at both the genetic and molecular levels. Mastreviruses of ∼2.8 kb in size encode 2 proteins each from virion and complementary strands, which are separated by long and short intergenic regions (Boulton, 2002; Brown et al., 2012). Virion strand-encoded proteins are necessary for viral movement and encapsidation (the enclosure of the viral DNA within the capsid), whereas replication-associated proteins are encoded in the complementary strand. The *Begomovirus* genus is the largest in the *Geminiviridae* family (comprising ∼380 virus species) and is subclassified into monopartite begomoviruses (containing a DNA A-like genome) and bipartite (with DNA A and DNA B genomes) begomoviruses (Figure 1). These monopartite and bipartite begomoviruses are mostly distributed in Old World (OW) and New World (NW) countries, respectively, with a few exceptions (Nawaz-ul-Rehman and Fauquet, 2009). Begomoviruses code for 5–8 ORFs through the generation of several overlapping viral transcripts under the control of promoters generally located within the intergenic region (Borah et al., 2016). The DNA A component of OW begomoviruses encodes 6 proteins: 4 in the complementary strand (C1–C4) and 2 in the virion strand (V1-V2) (Brown et al., 2012). Two genes in the virion strand encode for a coat protein (V1) and a pre-coat protein (V2) that aid in intra- and inter-cellular macromolecular movement, encapsidation, and insect transmission for plant-to-plant transport (Rojas et al., 1998, 2001; Liu et al., 1999). Four genes in the complementary strand encode a replication-associated protein (C1; Rep), a transcription activator protein (C2; TrAP), a replication enhancer protein (C3; REn) and a C4 protein (Brown et al., 2012). However, the DNA A component of NW bipartite begomoviruses lacks a V2 ORF and hence depends entirely on DNA B-encoded proteins for virus transport and systemic infection (Rojas et al., 2005; Brown et al., 2012). The DNA B of bipartite viruses encodes a movement protein (BC1; MP) and a nuclear shuttle protein (BV1; NSP) to facilitate virus-trafficking processes within and across cells (Sanderfoot and Lazarowitz, 1996; Ward and Lazarowitz, 1999).

The OW monopartite begomoviruses are often associated with satellite molecules such as alphasatellites and betasatellites (Nawaz-ul-Rehman and Fauquet, 2009; Kumar et al., 2017; Gnanasekaran et al., 2019). These alphasatellites and betasatellites are half the size (∼1.3 kb) of helper components and encode a single gene in the virion and complementary strand, respectively. Betasatellites require helper begomoviruses for their replication, encapsidation, insect transmission and systemic spread (Briddon and Stanley, 2006). Betasatellite-encoded βC1 proteins have been reported as pathogenicity determinants and perturb several host processes (Gnanasekaran et al., 2019). Moreover, the binding of Rep protein to a repeated DNA sequence motif (iteron) and an iteron-like sequence located at the replication origin of the DNA B and the betasatellite, respectively, is necessary to initiate their replication. However, unlike with DNA B, a more relaxed method of betasatellite trans-replication by helper begomoviruses has been demonstrated (Nawaz-ul-Rehman and Fauquet, 2009; Gnanasekaran et al., 2019).

Infectious geminivirus virions (22 × 38 nm in size) have two (geminate) incomplete T = 1 icosahedral capsid particles containing viral coat proteins, and each coat protein is bound by 7 bases of viral ssDNA (Hesketh et al., 2018). Once the viral genome is released from the capsid, it enters the cytoplasm of the infected cell and subsequently enters the cell nucleus, where it undergoes rolling-circle and recombination-dependent replication (Gutierrez, 1999). These newly replicated viral ssDNAs can be (1) converted into dsDNAs which can act as templates for another round of replication or transcription, (2) wrapped by viral movement proteins for transportation from the infected cell to adjacent cells through plasmodesmata (PD), or (3) encapsidated into infectious virions for long-distance virus transmission (Gutierrez, 1999; Hanley-Bowdoin et al., 2013). Moreover, geminiviruses possess a limited coding potential and thus rely heavily on host proteins to complete their infection cycle. They depend on host enzymes for their replication and transcription processes, coordinate with several cellular mechanisms to modulate cell division and the cell cycle, and manipulate host components at different cellular levels (Hanley-Bowdoin et al., 2013). Additionally, they encode multiple proteins that impair RNA silencing mechanisms by preventing small RNA generation and suppress several components of transcriptional gene silencing (TGS) and post-transcriptional gene silencing (PTGS) (Raja et al., 2010; Hanley-Bowdoin et al., 2013; Gnanasekaran et al., 2019; Prasad et al., 2019). These viral proteins also exploit RNA silencing machinery to contribute symptom remission in different crops and viruses (Ceniceros-Ojeda et al., 2016; Basu et al., 2018). This review summarizes the progress made in our understanding of geminivirus movement and its interactions with whiteflies for transmission. It documents the current perspective of the molecular arms race between geminiviruses and plants during the infection cycle. It also provides insights into the recent advancements made in the development of novel antiviral strategies and the ways that geminiviruses counteract them.

## Intracellular and Intercellular Movement of Geminiviruses

### Cellular Movement of Monopartite Viral Genomes

**FIGURE 2:**
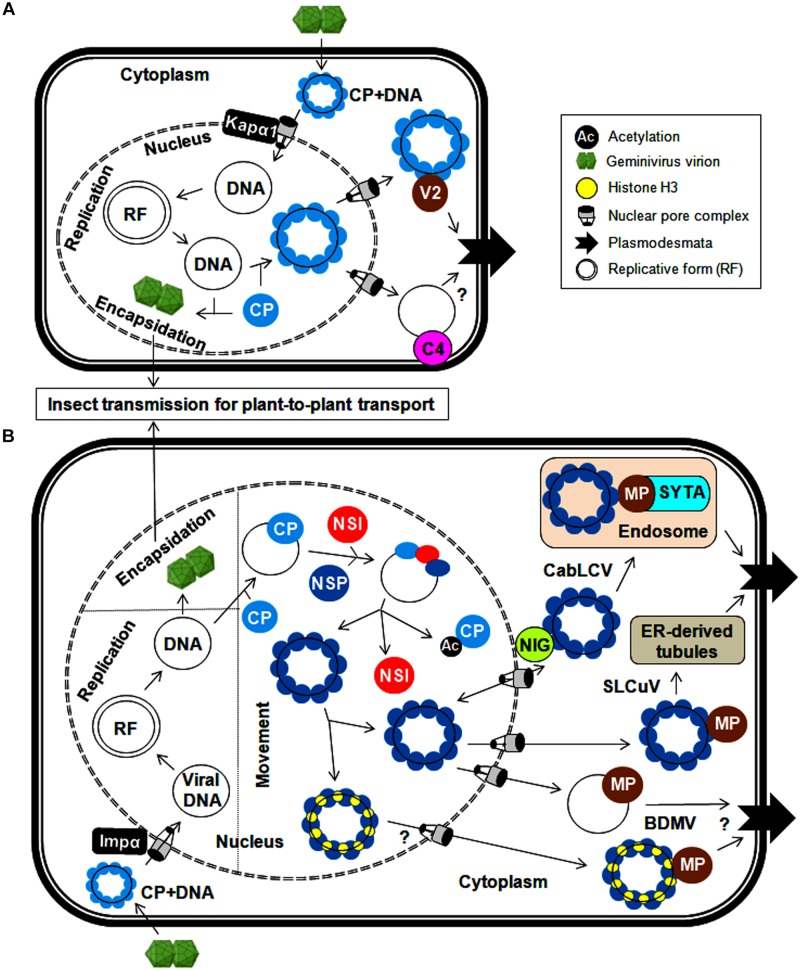
Cellular trafficking of geminiviral replicated genomes within and between plant cells for **(A)** monopartite geminiviruses and **(B)** bipartite geminiviruses. In the infected cells, circular geminiviruses replicate via a dsDNA intermediate, a replicative form of the viral genome. The CP is needed for the export of viral DNA from the nucleus and encapsidation for systemic spread. **(A)** In monopartite geminiviruses, the CP-bound viral genome (after uncoating) is imported from the cytoplasm to the nucleus by Kapα1 via nuclear pore complex. After replication, the CP binds to newly synthesized viral DNA and facilitates the export of viral genomes to the cytoplasm. For trafficking between cells, the V2 (likely C4) protein binds to this viral DNA-CP complex and traffics it through the endoplasmic reticulum to dock at the PD in the PM. **(B)** The viral genome-CP complex of bipartite begomoviruses is imported from the cytoplasm to the nucleus by Impα, and nucleo-cytoplasmic shuttling of viral DNA is mediated by a NSP and nuclear envelope-associated NIG. Meanwhile, NSI acetylates the CP and reduces its DNA-binding affinity, thereby assisting the binding of NSP to viral DNA. The MP (along with NSP) plays a crucial role in the transfer of viral genomes to the PD via endosomes by interacting with SYTA (CabLCV) or ER-tubules (SLCuV). The interaction of histone H3 with the NSP and MP (BDMV) also possibly facilitates this cell-to-cell transport.

Geminiviruses have developed various mechanisms for transporting uncoated viral genomes from the cytoplasm to the nucleus of the infected cell and transporting replicated viral genomes from the infected cell to neighboring cells. As coat protein (CP) is the only structural protein in the geminiviral capsid, it is necessary for viral capsid assembly. In addition, CP also plays a crucial role in viral DNA transportation by interacting with cellular transporters as exemplified for monopartite geminiviruses (Boulton et al., 1989; Padidam et al., 1996; Liu et al., 1997, 1999, 2001; Rojas et al., 2001; Sharma and Ikegami, 2009). Upon virus infection, the uncoating of viral genomes takes place in the cytoplasm, and the entry of viral ssDNAs into the nucleus is subsequently facilitated by the DNA-bound CP. Moreover, the CPs of several monopartite geminiviruses possess a nuclear localization signal (NLS) and a leucine-rich nuclear export signal (NES) and are thus localized to the nucleus (especially nucleolus), cytoplasm and cell periphery (Kunik et al., 1998; Ward and Lazarowitz, 1999; Unseld et al., 2001). In the nucleus, the viral ssDNAs are converted into dsDNAs through rolling-circle replication. These dsDNAs can then serve as templates for further replication/transcription. The CP of the maize streak virus (MSV) and the tomato yellow leaf curl Thailand virus (TYLCTHV) has been shown to bind non-specifically to ss and dsDNAs in a cooperative manner and sequester ssDNAs produced in the replication cycle (Liu et al., 1997; Pitaksutheepong et al., 2007). The binding of tomato yellow leaf curl virus (TYLCV)-CP with ssDNA resists nucleolytic cleavage of the viral genome, which can be encapsidated to form virions for long-distance plant-to-plant transport by whiteflies (Palanichelvam et al., 1998). However, the detailed processes involving virion packaging and preferences for ssDNAs over dsDNAs for encapsidation are inadequately investigated. The involvement of CPs in the shuttling of the CP-DNA complex from the nucleus to the cytoplasm has been elucidated (Figure 2A). Moreover, the interaction of the bhendi yellow vein betasatellite (BYVB)-βC1 protein and the TYLCV-CP with karyopherin α1 (Kapα1, a cellular transporter) may assist in nucleo-cytoplasmic trafficking (Kunik et al., 1999; Kumar et al., 2006). The involvement of the CP in this transportation mechanism makes it a functional replacement of NSP (in bipartite begomoviruses) for virus trafficking (Qin et al., 1998). However, none of the proteins encoded by monopartite geminiviruses have transporting functions similar to those of the DNA B-encoded movement protein. Recently, the phosphorylated and myristoylated C4 protein of a monopartite begomovirus (tomato yellow leaf curl China virus-TYLCCNV) has been shown to interact with a nuclear cargo protein (exportin α, XPO I) to function as a nuclear exporter (Mei et al., 2018a). However, concerted efforts are needed to explore the possible utilization of this C4-mediated process in virus movement, which may reveal insights into the molecular mechanism behind the movement of monopartite viruses.

Conversely, detailed mechanistic knowledge of how viral proteins coordinate the inter-cellular movement of monopartite begomoviruses is limited. The cell-to-cell movement of monopartite begomoviruses (TYLCV and cotton leaf curl Kokhran virus-CLCuKoV) is presumed to be through their interactions with movement proteins (V2 and/or C4) (Rojas et al., 2001; Priyadarshini et al., 2011). The V2 protein is targeted to the nuclear periphery and cytoplasm, and also co-localizes with the endoplasmic reticulum (ER). Similarly, the V3 protein of the *Beet curly top virus* (BCTV) functions as a movement protein and also co-localizes with the ER, which implies a possible viral infection route through the ER (Hormuzdi and Bisaro, 1995). In addition, the TYLCV-V2 protein forms cytoskeleton-dependent large aggregates, co-localizes with the CP, and binds to ssDNAs (Moshe et al., 2015). However, no experimental evidence illustrating the direct participation of the cytoskeleton in this V2-CP mediated cellular trafficking is available. Additionally, the ability of the BCTV-C4 protein to non-specifically bind to ss and dsDNAs and the localization of the East Africa cassava mosaic Malawi virus (EACMMV)-AC4 protein to the cell periphery through *N*-myristoylation make the C4/AC4 protein an ideal candidate for transporting viral DNAs to the cell periphery (Fondong et al., 2007; Teng et al., 2010). The MP of the MSV does not bind to DNA, so the CP alone is responsible for the entirety of these virus-trafficking events (Kotlitzky et al., 2000; Liu et al., 2001). In the presence of the C4 protein, an increase in the efficiency of TYLCV movement to adjacent cells by the V2 protein has also been observed (Rojas et al., 2001). Thus, C4 and V2 proteins cooperate with the CP in the inter-cellular movement of monopartite geminiviruses through PD by increasing their size-exclusion limit (Figure 2A). Intriguingly, a possible impermeable boundary blocking the movement of phloem-limited geminiviruses from infected phloem to nearby non-phloem cells needs to be further investigated. Nonetheless, the role of V2 and C4 proteins in maintaining/breaking this barrier can be analyzed by expressing them under phloem-specific promoters.

### Trafficking of Bipartite Begomoviruses

The DNA B of bipartite begomoviruses encodes two proteins (NSP and MP) for exclusive transport of viral DNA within and between cells (Noueiry et al., 1994; Sanderfoot and Lazarowitz, 1996; Sanderfoot et al., 1996; Ward et al., 1997). Similar to the mechanism in their monopartite counterparts, after the uncoating of the viral genome in the cytoplasm, the viral DNA-bound CP of bipartite begomoviruses is redirected to the nucleus, particularly the nucleolus, via its NLS (squash leaf curl virus, SLCuV-CP) at the N-terminus and via interactions with importin α, a nuclear transporter (mungbean yellow mosaic India virus, MYMIV-CP) (Qin et al., 1998; Guerra-Peraza et al., 2005). After the completion of virus replication, the SLCuV-CP binds non-specifically to viral ssDNAs and sequesters replicated viral genomes away from the replication site for viral encapsidation and long-distance movement or transport to neighboring cells (Pascal et al., 1994). However, the CP of bipartite begomoviruses is not involved in virus movement. For nucleo-cytoplasmic trafficking, the *Cabbage leaf curl virus* (CabLCV)-NSP recruits an acetyltransferase (NSI) to cooperate in this NSP-mediated process (McGarry et al., 2003). The NSI can form a higher-order oligomeric enzyme complex and acetylate plant proteins. However, the DNA-unbound NSP inhibits the self-aggregation and enzymatic activity of NSI, resulting in the recruitment of NSI monomers to the CP-DNA complex (Figure 2B). These monomeric NSIs acetylate the DNA-bound CP, thereby reducing its DNA-binding affinity (Carvalho et al., 2006). The NSP then binds to viral DNA, facilitating the transport of the viral DNA-NSP complex between the nucleus and cytoplasm (Ward and Lazarowitz, 1999). The redirection of the viral DNA-NSP complex from the nucleus to the cytoplasm is mediated by cytoplasmic GTPase (NIG) and/or histone H3. This NIG possesses intrinsic GTPase activity and accumulates around the nuclear envelope (Figure 2B). Therefore, the interaction of the NSP (CabLCV, *Tomato golden mosaic virus*-TGMV and *Chino del tomate virus-Tomato*-CdTV) with NIG forms a viral DNA-NSP-NIG complex and helps translocate the viral DNA-NSP complex from the nucleus to cytoplasm of the infected cell (Carvalho et al., 2008). This NSP-NIG interaction also facilitates the re-entry of viral DNA from the cytoplasm to the nucleus of neighboring cells for new rounds of virus replication. Alternatively, both the NSP and MP of the *bean dwarf mosaic virus* (BDMV) bind to histone H3, forming a highly compact viral DNA-H3-NSP-MP complex in the nucleus that facilitates the transport of the viral movement complex to the cell periphery and PD (Zhou et al., 2011).

The cell-to-cell movement of phloem-limited SLCuV is interesting because SLCuV-MP did not bind ssDNA or dsDNA and so SLCuV-NSP facilitates the cell-to-cell virus movement by preferentially binding to viral ssDNA (Pascal et al., 1994). The N-terminus of the SLCuV-NSP is necessary for the formation of this ssDNA-NSP complex in the nucleus, whereas the C-terminus of the NSP associates with the MP to facilitate systemic movement of viral DNA (Sanderfoot et al., 1996). Further, the SLCuV-MP co-localizes with virus-induced ER-derived tubules that extend across the walls of procambial cells in the systemic leaves of pumpkin plants (Ward et al., 1997). The *Abutilon mosaic virus* (AbMV)-MP exploits cellular membrane flow to transfer the complex from the ER to PD and into adjacent cells (Zhang et al., 2002). Hence geminiviruses employ the ER as a conduit for cell-to-cell trafficking of the viral genome to PD at the plasma membrane (PM) (Figure 2B). In mesophyll cells, the BDMV-MP moves between cells, modifies size-exclusion limit of PD and mediates the cell-to-cell movement of viral DNA (Noueiry et al., 1994). Also both NSP and MP of BDMV bind dsDNA and so it appears that there is an exchange of viral dsDNA from the NSP to the MP for cell-to-cell transfer of BDMV DNA (Rojas et al., 1998). Apparently, phloem-limited and phloem-mediated geminiviruses may have evolved distinct mechanism for their cell-to-cell movement.

The MP also interacts with host proteins involved in vesicular transport to assist virus movement. One of these host proteins is synaptotagmin (SYTA), which regulates synaptic vesicle endocytosis and endosome trafficking (Lewis and Lazarowitz, 2010). The MP of CabLCV associates with SYTA at the PM, which leads to redirection of the viral movement complex to early endosomes. These endosomes can mobilize and dock the complex at the PD via a recapture pathway for cell–cell virus movement. Thus, the interaction between the MP and SYTA provides evidence for the vital role of plant endocytic recycling pathways in transporting the MP to PD (Figure 2B). Likewise, co-localization of the AbMV-MP with PM-localized stomatal cytokinesis defective protein 2 (SCD2) may possibly help in cell-cell movement of viruses (Krapp et al., 2017). Using a heterologous system, the possible involvement of microtubules in begomovirus infection may be tested by the detection of MP-SCD2 interactions. However, extensive experimentation is needed to identify new potential plant protein interactions and comprehensively substantiate the involvement of the cytoskeleton and/or microtubule network in assisting virus cellular transport. In addition, the AbMV-MP viral movement complex associates with a heat shock cognate (70-kDa protein) which facilitates movement through stromules and plastids (Krenz et al., 2012). These studies indicate that geminiviruses exploit multiple macromolecular trafficking routes for intra- and inter-cellular transportation of infectious viral forms. Finally, these viral MPs increase the size exclusion limit of the PD of the infected cell and assist the entry of viral DNA-NSP complex into the cytoplasm of the adjacent cells. The ability of nucleo-cytoplasmic shuttling of NSP facilitates the entry of viral DNA from the cytoplasm into the nucleus of adjacent cell for a fresh round of virus replication.

## The Tug-Of-War Between Geminiviruses and Hosts

The study of geminiviruses at the molecular level is interesting due to their ability to manipulate host responses despite their limited genetic coding capacity. Geminiviral proteins are required for viral replication, transcription, encapsidation, transport, and vector transmission. However, they recruit and interact with a wide range of host proteins and manipulate several cellular mechanisms to perform these functions. To defend against geminivirus infections, plants also develop and modify antiviral approaches involving novel host proteins at multiple stages of infection. Nevertheless, viruses counteract these host-mediated defense strategies by employing dynamic and coordinated responses.

### Cell Cycle Regulation

**FIGURE 3:**
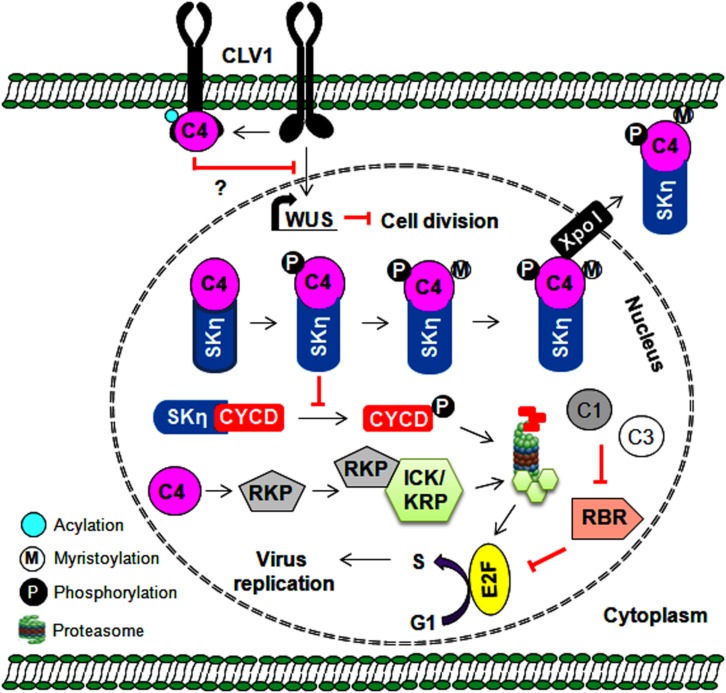
Multiple roles of C4 protein in regulating the cell cycle and cell division. The C4 protein reprograms the cell cycle either by preventing SKη-mediated degradation of CYCD or by probably degrading cell cycle inhibitors (ICK/KRP). The C4 protein also indirectly regulates cell division by interfering with CLV1-mediated expression of the WUS gene. Further, the expression of WUS gene is reported to inhibit cell division. Thus, by regulating the cell cycle/cell division, the C4 protein provides a favorable environment for virus replication in infected plant cells.

Geminiviruses depend exclusively on host polymerases and associated factors to synthesize new viral DNA in association with Rep and REn proteins. These viruses infect differentiated cells when the cell cycle is arrested to prevent additional rounds of plant genome replication. During infection, it is necessary for geminiviral proteins to redirect these cell types and form essential replication-associated complexes to assist with virus multiplication (Gutierrez, 1999). The Rep and REn proteins of several geminiviruses interact with RBR (retinoblastoma-related protein) to redirect the cell cycle via E2F-dependent transcription of cell cycle regulators (Gutierrez et al., 2004) (Figure 3). These viral proteins recruit plant DNA polymerases and interact with several host factors, including proliferating cell nuclear antigen (PCNA), replication factor C, and minichromosome maintenance protein 2, to assist with virus replication (Hanley-Bowdoin et al., 2013). AC4/C4 proteins of several geminiviruses also manipulate various cell differentiation regulators (such as ATHB7, ATHB12, CYCD1;1, and TRN1), which leads to ectopic cell division and expansion of the infected cells (Lai et al., 2009; Mills-Lujan and Deom, 2010; Mandal et al., 2015; Mei et al., 2018b). In addition, AC4/C4 proteins either prevent or mediate the degradation of cell cycle regulators and are important to the establishment of a permissive environment for virus amplification. For example, BCTV-C4 induces the expression of a RING finger protein (RKP) that interacts with and possibly degrades cyclin kinase inhibitors (ICK/KRP) to promote mitotic cycles (Lai et al., 2009). Furthermore, the interaction of TYLCCNV-C4 with shaggy-like kinases η (SKη) helps target SKs to the PM and impedes the phosphorylation-mediated degradation of its target, CYCD1;1 (Figure 3). This increased stability of CYCD1:1 results in the induction of abnormal cell division in the infected plant cell (Mei et al., 2018a, b). Moreover, membrane-bound CLAVATA1 (CLV1) represses the expression of WUSCHEL (WUS) to regulate cell differentiation in shoot meristem maintenance. The interaction of TYLCV-C4 (*S*-acylation dependent) with CLV1 and the inhibition of WUS by C4 proteins (Li et al., 2018) suggest that C4 proteins may interfere with this CLV1 pathway to induce ectopic cell division (Figure 3).

In addition, geminiviruses interact with hormones that regulate cell differentiation and cell division. For instance, the inactivation of adenosine kinase 2 (ADK) by TrAPs of BCTV and TGMV (Wang et al., 2005) suggests a possible relationship between geminiviruses and cytokinin responses. ADK converts cytokinin nucleosides into low-activity nucleotide forms and contributes to plant cytokinin homeostasis (Schoor et al., 2011). The TrAP-ADK interaction-dependent elevation of several cytokinin-responsive genes, such as ARR5 and ARR16, has been reported during geminivirus infections (Baliji et al., 2010). Therefore, it would be interesting to study the role of the TrAP/ADK/SnRK1 (Sucrose non-fermenting 1-related protein kinase 1) complex in modulating cell cycle regulation and promoting virus proliferation. Geminivirus infection also induces the expression of a DNA-binding protein (TIFY4B) that acts as a geminiviral resistance factor. However, the interaction of CabLCV and TGMV TrAPs with TIFY4B inhibits its potential role in cell cycle arrest (Chung and Sunter, 2014). Thus, geminiviruses utilize the replication machinery of the infected host and employ multiple strategies to regulate cell division and the cell cycle. This consequently facilitates viral replication and the progression of endoreduplication cycles.

### Inhibition of Cell Death Response

To successfully propagate in the infected cells, it is vital for viruses to impede host cellular defense pathways. Geminivirus infection induces the expression of senescence- and cell death/hypertensive response (HR)-related transcripts without inducing a visible cell death phenotype (Ascencio-Ibáñez et al., 2008). In tomatoes, TYLCV infection impairs the cell death response mediated by heat shock protein 90 (HSP90) and suppressor of the G2 allele of Skp1 (SGT1) (Moshe et al., 2016). This suggests that some geminiviral proteins can perturb the cell death response. However, the mechanism behind this activity is unclear. To counter the HR, the TYLCV-V2 protein likely interacts with a papain-like cysteine protease and inhibits its enzymatic activity (Bar-Ziv et al., 2015). Furthermore, some geminivirus-encoded proteins (Rep, NSP, V2) are known to induce the HR (Garrido-Ramirez et al., 2000; Amin et al., 2011). However, the TrAPs of some monopartite begomoviruses counter the cell death induced by the V2 protein (Mubin et al., 2010). Moreover, the interactions of SnRK1 with TrAP and its role in cell death are well-known (Hao et al., 2003; Hulsmans et al., 2016). Therefore, SnRK1 could probably associate with TrAP in countering the HR, but such mechanisms require further exploration. Furthermore, tomato yellow leaf curl China betasatellite (TYLCCNB)-βC1 interacts with two protein kinases (mitogen-activated protein kinase 4-MAPK4 and MAPK kinase 2-MKK2) involved in host MAPK pathway and inhibits its kinase activity (Hu et al., 2019). However, the mechanistic details behind this MAPK cascade interference by viral protein(s) to counter host cell death mechanism need to be explored.

### Misregulation of Plant Methyl Cycle

In addition to its role as a nutritional factor limiting protein synthesis, methionine is essential for epigenetic modifications because it donates methyl groups to DNA. These methyl groups (an essential component necessary for cytosine methylation) are generated through the methyl cycle in plants. However, geminiviruses efficiently impede cytosine methylation of the viral genome through multiple viral proteins, which act at different stages of the methyl cycle. For example, TYLCCNB-βC1 binds to *S*-adenosyl homocysteine hydrolase (SAHH), a methyl cycle-associated enzyme that is required for *S*-adenosyl methionine (SAM) production (Yang et al., 2011). BCTV-TrAP interacts with and attenuates the degradation of *S*-adenosyl methionine decarboxylase 1 (SAMDC1), thereby preventing the conversion of SAM to dcSAM (Zhang et al., 2011). Similarly, SAM synthetase (SAMS), an enzyme that adenylates methionine to SAM, is inhibited through interactions with the cotton leaf curl Multan virus (CLCuMuV)-encoded C4 protein (Ismayil et al., 2018). It is evident from these observations that plants incorporate the methyl cycle as a vital link in defending against DNA viruses. Importantly, inhibition of the methyl cycle by geminiviruses poses a serious challenge to the engineering of RNA silencing-mediated virus resistance.

## Altered Host Gene Expression and Hormonal Signaling

During infection, several plant genes/proteins associated with the development and defense responses including transcription factors such as bHLH, NAC, MYC, PEAPOD2, WRKY, and MYB are differentially regulated (Ascencio-Ibáñez et al., 2008; Sahu et al., 2010; Wang et al., 2015; Huang et al., 2016, 2017). Furthermore, CabLCV-NSP induces and redirects ASYMMETRIC LEAVES (AS) 2 from the nucleus to the cytoplasm to accelerate the decapping activity of DCP2, which leads to indirect inhibition of virus-derived small RNA accumulation (Ye et al., 2015). Therefore, differential regulation of various host factors upon infection renders infected plants more susceptible to geminiviruses.

Several geminivirus-encoded proteins interact with various host-derived proteins to assist with virus replication, cellular movement, and insect transmission. However, these interactions also obstruct plant hormone biosynthesis or signaling. Plant hormones are the major regulators of homeostatic balance between development and stress, so they can be exploited by viruses to overcome plant defense responses. Transcripts of host defense response hormones (such as ethylene, jasmonic acid, and salicylic acid) are generally suppressed during geminivirus infections because activation of these genes affects virus infection (Ascencio-Ibáñez et al., 2008; Chen et al., 2010). In addition, transient or stable expression of some TrAPs alters the expression of several genes involved in sugar and jasmonate-responsive pathways (Lozano-Duran et al., 2011; Soitamo et al., 2012). However, the biological consequences of such changes have not yet been characterized. Furthermore, the tomato yellow leaf curl China betasatellite (TYLCCNB)-βC1 protein oligomerizes with host AS1, a transcription factor involved in leaf development. As a consequence, TYLCCNB-βC1 antagonizes AS1/AS2 heterodimerization and modulates the expression of several leaf-specific and jasmonic acid-responsive transcripts, which results in betasatellite infection-like phenotypes (Yang et al., 2008).

Brassinosteroid (BR) hormone plays a vital role in plant development and defense responses by forming a cooperative network with other plant hormonal pathways (Belkhadir and Jaillais, 2015). Altered expression of BR-responsive genes was noticed in C4 over-expressing plants, which suggests the direct role of C4 in modulating the BR pathway. Several AC4/C4 proteins interact with multiple shaggy-like protein kinases, a central negative regulator of the BR pathway (Piroux et al., 2007; Mills-Lujan and Deom, 2010). Both C4- and BR-specific transcription factors (BES1 and/or BZR1) are targeted by SKs, so C4 interacts with SKs and makes itself a substrate for phosphorylation, which subsequently allows entry of the hypo-phosphorylated BES1/BZR1 protein to the nucleus (Piroux et al., 2007). This nucleus-redirected BES1/BZR1 transcription factor down-regulates the expression of several genes involved in anther and pollen development and affects male fertility (Bi et al., 2017). These observations support the idea that geminivirus AC4/C4 proteins exploit BR-mediated plant kinases affecting male fertility and regulate cell division (as mentioned above) to promote early to late stages of virus infection.

## Utilizing Post-Translational Modification System

**FIGURE 4:**
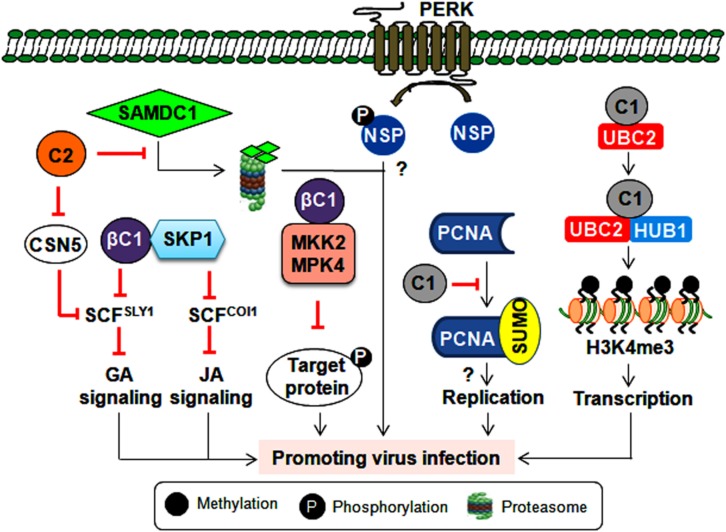
Consequence of post-translational modification of viral and host proteins during geminiviral pathogenesis. Virus-encoded TrAP inhibits plant defense responses either by modulating the derubylation of CUL1 (removal of RUB, a ubiquitin-like protein, from CULLIN in the active E3 ubiquitin ligase complex inactivates its E3 ligase activity) or by attenuating the degradation of the SAMDC1 enzyme. However, the βC1 protein impairs the ubiquitin degradation pathway and hormonal (jasmonic acid and giberellic acid) signaling by interacting with the E2 conjugating enzyme (UBC3) and SKP1, respectively. Also by inhibiting the kinase activity of MPK4 and MKK2, βC1 protein can possibly prevent the phosphorylation of the target proteins. Furthermore, the NSP is phosphorylated by PERK, which potentiates virus infection by an unknown mechanism. The C1 protein probably helps with virus replication by modulating the SUMOylation of PCNA. Additionally, the C1 protein interacts with histone mono-ubiquitination machinery (UBC2 and HUB1) to facilitate the transcription of viral genes.

Every aspect of the plant life cycle is governed by the regulated synthesis of new proteins and the coordinated elimination of unwanted proteins. These proteins also undergo post-translational modifications (PTMs) such as phosphorylation, ubiquitination, and SUMOylation (He et al., 2017). Several geminiviral proteins are known to interact/interfere with these machineries to alter host-mediated defense responses and create a favorable environment for successful initiation and completion of the infection cycle. One such scenario is the phosphorylation of NSP (of CabLCV, TGMV and CdTV) by a proline-rich extensin-like receptor kinase to function as a pro-viral host factor for these bipartite begomoviruses (Florentino et al., 2006) (Figure 4).

### Manipulation of Ubiquitin-Proteasomal Pathway

The linkage of the ubiquitin chain to the lysine residue of target proteins by E1, E2, and E3 ubiquitin ligase complex leads to proteasomal-mediated degradation of poly-ubiquitylated target proteins. As poly-ubiquitylation of host proteins regulates several key cellular processes, it is imperative for geminiviruses to alter this ubiquitin machinery at multiple stages to complete their infection cycle. The interaction of CLCuMuB-βC1 with the host E2 ubiquitin-conjugating enzyme (UBC3) culminates in reduced global poly-ubiquitination of proteins upon βC1 overexpression (Eini et al., 2009). In infected plants, this interaction helps in inducing betasatellite-specific symptoms possibly by perturbing several developmental and hormonal pathways. In addition, geminivirus infections impair SCF (SKP1-Cul1-F box)-based E3 ubiquitin ligase complexes to modulate different hormonal signaling events. For instance, several TrAPs bind to the CSN5 of the COP9 signalosome and alter the derubylation of the CUL1 of the SCF complex (Lozano-Duran et al., 2011). This results in the impairment of the SCF^SLY1^ complex, which leads to the differential response of giberellic acid signaling during infection (Lozano-Duran et al., 2011). Alternatively, the cotton leaf curl Multan betasatellite (CLCuMuB) encoded-βC1 protein interacts with S-phase kinase-associated protein 1 (SKP1) and affects the assembly of SCF-based ligase complexes (Figure 4). This interaction hinders the biological function of SCF^COI1^ and SCF^SYL1^ complexes involving jasmonic acid and gibberellic acid pathways, respectively (Jia et al., 2016).

Geminiviruses also interfere with host ubiquitin proteasomal machinery to regulate the cell cycle or viral transcription (Figure 4). The Rep protein of a chilli leaf curl virus (ChiLCV) interacts with histone H2B monoubquitination machinery (UBC2 and HUB1) to promote H3K4me3 on viral chromatin, which results in the enhanced transcription of viral genes (Kushwaha et al., 2017). Moreover, BCTV-TrAP impedes TGS by interacting with SAMDC1 to attenuate its proteasome-mediated degradation (Zhang et al., 2011). These interactions may create a permissive cellular environment for virus replication and/or transcription. Interestingly, to counter virus infection, plants also exploit ubiquitin-proteasomal degradation to target virus-encoded virulence factors. For instance, a tobacco-encoded RING finger protein mediates the degradation of the CLCuMuB-βC1 protein through the ubiquitin proteasomal pathway (Shen et al., 2016). Thus, geminiviruses compete with host plants to subvert ubiquitin-mediated protein degradation and circumvent host defense responses. Importantly, unraveling the PTM-related mechanisms behind antiviral plant defense strategies may provide a guiding tool for designing virus resistance strategies that can protect several economically important crops.

### Modulation of Host SUMOylation

The conjugation of small ubiquitin-like modifiers (SUMO) is regarded as one of the crucial links in maintaining plant growth, development and defense equilibrium (He et al., 2017). TYLCV-Rep has been demonstrated to bind to the E2 SUMO conjugating enzyme (SCE-1), which conjugates SUMO to target proteins and helps in virus replication (Sanchez-Duran et al., 2011). However, the SUMOylation state of global host proteins is unaltered during ectopic expression of the Rep protein, implying that the interaction between Rep and SCE-1 results in the SUMOylation of very specific host proteins. One such host protein involved in virus multiplication is PCNA, which controls the cell cycle, DNA replication, and DNA repair. PCNA switches between these biological functions by undergoing post-translational modifications. For example, tomato PCNA is SUMOylated at two different lysine acceptor sites. Hence, TGMV-Rep interacts with PCNA and impedes its SUMOylation, thereby switching its cellular processes to possibly assist with virus replication (Arroyo-Mateos et al., 2018). These findings further underscore the efficient utilization of SUMOylation to aid virus infection (Figure 4). However, exactly how this SUMO modulation of host factors helps in promoting virus propagation is unknown. Thus, identifying key plant SUMO targets could allow us to decode the signaling cascade involved in viral susceptibility and/or resistance. Moreover, several chromatin remodelers and defense-related transcription factors are also known SUMO targets (He et al., 2017). Therefore, exploring the complexity of SUMO-dependent cellular crosstalk may reveal new ways to uncouple the trade-off between plant growth and defense responses.

## Combating Host-Mediated Antiviral Strategies

### Cellular SnRK1-Mediated Defense Regulation

**FIGURE 5:**
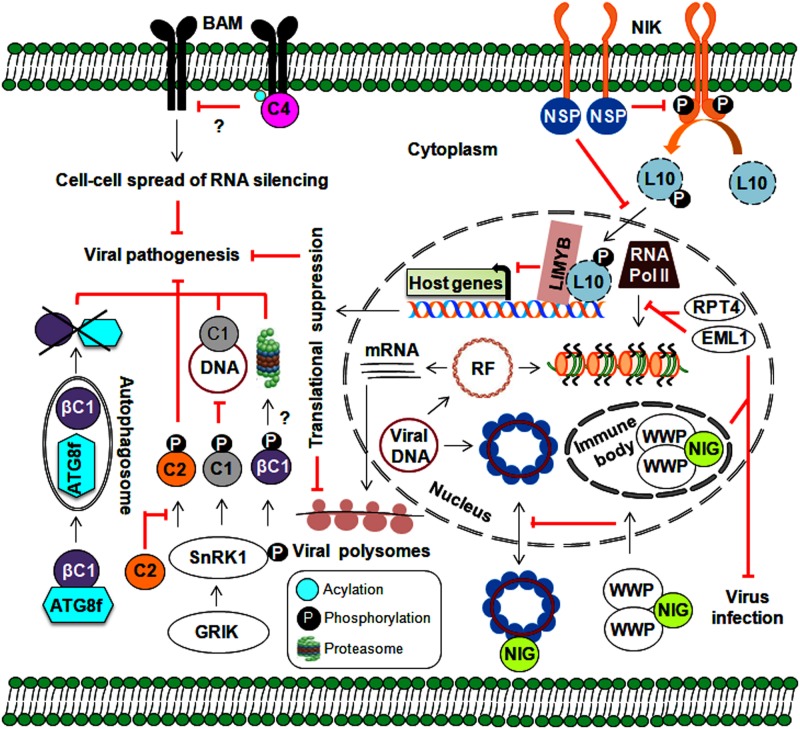
Implication of various approaches employed by host-mediated resistance factors in defense against geminiviruses. Plants implicate protein kinases (GRIK1 and SnRK1) and an autophagy pathway in several stages of the geminivirus infection cycle. Moreover, plant nuclear-bound RPT4 and EML1 inhibit viral transcription by preventing the binding of RNA polymerase II to viral chromatin. Plant kinases also prevent the nucleo-cytoplasmic trafficking of viral genomes through the sequestering of NIG (by WWP) from the cytoplasm to nuclear immune bodies. The NIK-mediated antiviral strategy involves the formation of a phosphorylated L10-LIMYB complex resulting in global translational suppression and prevention of polysome binding to viral transcripts. Membrane-localized BAMs are also employed by plants to assist with the cell-to-cell spread of RNA silencing.

As a global central regulator of host metabolism, SnRK1 detects energy deficiencies in plants and plays a central role in regulating energy homeostasis during stress responses (Hulsmans et al., 2016). Geminivirus infection upregulates the expression of two plant SnRK1-activating kinases (Geminivirus Rep-interacting kinase-GRIK1 and 2), and these transcripts are increased during early infection. However, expression does not change during development (Shen and Hanley-Bowdoin, 2006). TGMV-Rep interacts with GRIK 1 and 2, possibly targeting them to the nucleus to alter plant development during infection (Kong and Hanley-Bowdoin, 2002). As functional serine/threonine kinases, GRIKs undergo autophosphorylation and also phosphorylate the catalytic subunits on the conserved T-loop of SnRK1 to activate its kinase activity (Shen et al., 2009). In turn, this activated SnRK1 phosphorylates TGMV-Rep at its overlapping DNA-binding and DNA-nicking domains (at ser-97), which results in compromised Rep binding to the viral genome (Shen et al., 2018). However, a phosphorylation-deficient mutant of ser-97 replicates efficiently in tobacco protoplasts. On the other hand, the TrAPs of BCTV and TGMV interact with SnRK1 through a conserved region and inhibit its kinase activity, thereby preventing its autophosphorylation (Hao et al., 2003). The phosphorylation of CabLCV-TrAP (at ser-109) by SnRK1 and the overexpression of SnRK1 lead to delayed CabLCV infection (Shen et al., 2014). Similarly, the SnRK1 protein interacts with and phosphorylates the TYLCCNB-βC1 protein, which may lead to the degradation of the βC1 protein and the attenuation of virus infection (Shen et al., 2011). Overexpression of SnRK1 seemingly induces the expression of several autophagy marker genes (Baena-Gonzalez et al., 2007), which suggests the possible interference of autophagy in this SnRK1-mediated geminiviral resistance mechanism (Figure 5). The TrAPs of several geminiviruses inactivate ADK, an enzyme that catalyzes 5′-AMP salvage synthesis and may control SnRK1 activity through AMP-mediated inhibition of catalytic subunit dephosphorylation (Wang et al., 2005). Furthermore, SnRK1 forms a complex with ADK in the cytoplasm, so any alterations in either one will result in the impaired activity of other kinases as well (Mohannath et al., 2014). This SnRK1 signaling cascade represents a new paradigm for primary host defenses against geminiviruses. However, the molecular mechanisms involved in the downstream target processes employed by SnRK1-phosphorylated viral proteins have not been clearly investigated. As SnRK1 plays a vital role in biotic interactions (Hulsmans et al., 2016), exploring the effects of this kinase on stress-response hormones (such as ethylene, jasmonic acid, and salicylic acid) will be useful to our understanding of the components involved in this defense response.

### Virus Suppression Through Autophagy or Calmodulin-Like Proteins

Several geminiviral TrAPs have been shown to induce the expression of the calmodulin-like protein (rgsCaM), a known regulator of RNA silencing machinery. They can sequester rgsCaM to the nucleus, possibly preventing its degradation (Chung et al., 2014). Similarly, the TYLCCNB-βC1 protein also induces the expression of rgs-CaM. Interestingly, to stop the generation of secondary siRNAs, rgs-CaM interferes with the RNA silencing pathway at two stages: (1) by reducing the expression of RNA-dependent RNA polymerase 6 (NbRDR6) and (2) by interacting with Suppressor of Gene Silencing 3 (NbSGS3), leading to its degradation through an autophagy pathway (Li F. et al., 2014; Li et al., 2017). Plants employ autophagy as a powerful antiviral tool and to maintain balance among cellular machinery (Choi et al., 2018). Consequently, geminiviruses can indirectly subvert this defense strategy for immune evasion via rgs-CaM. On the other hand, the direct involvement of autophagy in begomovirus infection comes from the interaction of the CLCuMuB-βC1 protein with a key autophagy-related NbATG8f protein, which results in the degradation of the βC1 protein (Haxim et al., 2017). Both overexpression of rgs-CaM and silencing of autophagy-related genes lead to enhanced susceptibility to geminivirus infection. Thus, autophagy plays an emergent role in geminivirus pathogenesis through the suppression of RNA silencing and the degradation of viral virulence factors (Figure 5). However, the exact mechanisms by which autophagy-related (ATG) genes govern autophagy during viral infection have not been fully uncovered. In animals, manipulation of innate immunity against viruses occurs via cross-talk between autophagy and several cellular components, such as pattern recognition receptors (PRRs) and macromolecular trafficking (Lee and Min, 2007). This necessitates a comprehensive analysis of research aimed at understanding the functional details about how plants incorporate autophagy into defense against geminiviruses.

### PM-Bound Receptor-Like Kinase-Mediated Immunity

In general, the first level of microbial recognition is carried out by PRR-mediated PAMP-triggered immunity (PTI). PRRs are membrane-bound receptor-like proteins/kinases (RLKs) involved in plant growth, development, and defense against numerous pathogens (Tang et al., 2017). It has been widely established that plants employ RNA silencing to defend against geminivirus infection. Thus, to hinder the cell-to-cell spread of RNA silencing, the TYLCV-encoded C4 protein interacts with BARELY ANY MERISTEM (BAM) 1 and 2, which are PM-localized RLKs (Rosas-Diaz et al., 2018). Although virus infection is known to elicit such events, understanding the underlying molecular aspects and how these RLKs stimulate downstream signaling events would allow us to uncover the exciting details behind this enhanced virulence.

An NSP-interacting GTPase (NIG) on the cytoplasmic side of the PM helps with nuclear export of viral DNA to the cytoplasm (Figure 5). However, WW domain-containing proteins (WWPs) relocate cytoplasmic NIG to immune nuclear bodies (NBs) in the nucleus, thereby affecting its nucleo-cytoplasmic trafficking capacity (Calil et al., 2018). By entrapping NIG in NBs, WWPs confer resistance to begomovirus infection. Deeper insights into the mechanism behind NB assembly may help us better understand how it functions as a defense layer and corresponding begomovirus counter-defenses.

Moreover, the interaction of CabLCV, TGMV, and CdTV NSPs with NSP-interacting kinase 1 (NIK1), a membrane receptor kinase, sheds additional light on the role of NSPs in virus pathogenicity (Fontes et al., 2004; Mariano et al., 2004). Upon the elicitation of NIK1 (which occurs through autophosphorylation at thr-469 and thr-474) by virus infection, RPL10 is indirectly phosphorylated by NIK1 and then translocated from the cytoplasm into the nucleus, which facilitates its interaction with the L10-interacting MYB domain-containing protein (LIMYB). This RPL10-LIMYB interaction leads to the translational inhibition of several plant genes and decreased viral transcript association with polysomes (Rocha et al., 2008; Zorzatto et al., 2015). To counteract this defensive signaling, NSP interacts with a kinase domain (A-loop), prevents the autophosphorylation of NIK1, and inhibits the activation of a downstream antiviral cascade (Santos et al., 2010). Furthermore, the overexpression of NIK1 attenuates symptom determination and impairs virus infection. However, by functioning as a negative immune regulator, NIK1 may also have an antagonistic effect on the translation of innate antiviral proteins, which could reduce the effectiveness of this as a defense strategy. Ligand-induced recruitment of co-receptors is essential for PRRs to attain functionality. This necessitates further study to uncover the entire NIK1-mediated network (including the identification of NIK1 co-receptors), which could help us devise NIK1-based antiviral resistance methods that do not compromise the innate immunity of the host.

### Chromatin-Associated Proteins as Viral Resistance Factors

The infection prevalence of multiple begomoviruses and their synergistic effects in the field have prompted geminivirologists to explore innate plant resistance genes that confer resistance/tolerance to these infections. Natural resistance factors (Ty-1 and Ty-3) against TYLCV infection that encode DFDGD-class RNA-dependent RNA polymerases have been introgressed into tomatoes (Verlaan et al., 2013). To overcome this Ty-derived resistance, a recombinant strain (TYLCV-IS76) has replaced the susceptible TYLCV parent strain and consequently causes disease in these Ty-resistant cultivars (Belabess et al., 2015). Thus, the evolution of geminiviruses through recombination helps replace milder strains, improves symptom severity, and facilitates the invasion of previously uninfected plants.

In tomatoes, tomato leaf curl New Delhi virus (ToLCNDV) infection induces the expression of an ATPase (RPT4) and a DEAD-box RNA helicase (DEAD35) (Sahu et al., 2010). This RPT4 binds to the intergenic region of the ToLCNDV genome and inhibits the transcription of viral genes by RNA polymerase II (Sahu et al., 2016). Furthermore, transient DEAD35-silencing in a tolerant cultivar results in increased susceptibility to ToLCNDV infection (Pandey et al., 2019). Similarly, *Arabidopsis* histone reader EML1 (EMSY-LIKE 1) suppresses CabLCV infection by inhibiting the association of RNA polymerase II with viral chromatin and represses viral gene expression (Coursey et al., 2018). Aside from SnRKs and RLKs, plant-derived chromatin modelers have been co-opted as viral resistance factors to defend against geminivirus infections (Figure 5).

## Interactions Facilitating Insect Transmission

**FIGURE 6:**
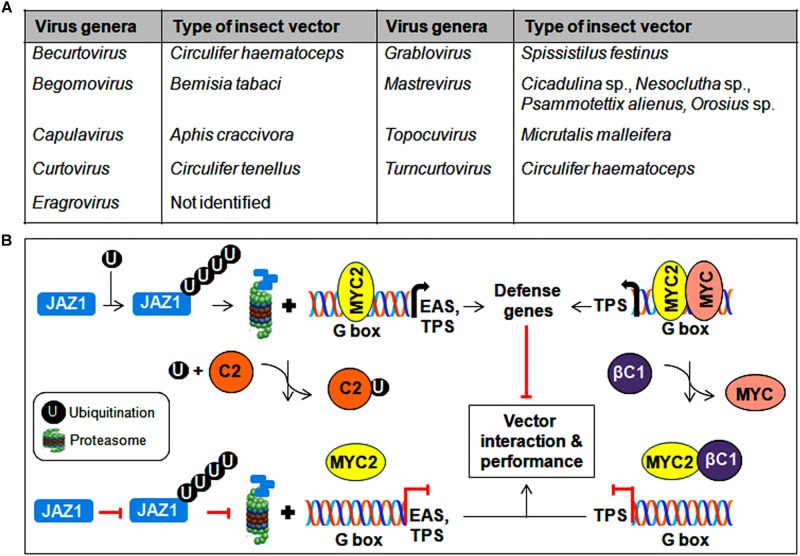
Insect vector-mediated transmission of geminiviruses. **(A)** List of different genera of the *Geminiviridae* family and its associated insect vectors. **(B)** Interference of MYC2-regulated gene expression by geminiviral proteins to modulate insect transmission. Begomovirus-encoded TrAP binds to ubiquitin and impedes the degradation of JAZ1, whereas the βC1 protein prevents MYC2 from binding to the G-box in the promoter of downstream target genes. As a consequence, the expression of defense-related genes is inhibited, resulting in the improved performance of the vector in virus transmission.

The mode of geminivirus transmission between plants is facilitated by various homopteran insects including aphids, leafhoppers, treehoppers, and whiteflies (Figure 6A). Among them, a circulative persistent manner of begomovirus transmission by the *Bemisia tabaci* (whitefly) is widely investigated (Czosnek et al., 2017). Virions from plant phloem were ingested by an insect subsequently travel along the food canal, foregut, and esophagus to get access to the early endosome of the vector midgut through the clathrin-dependent pathway. This early endosome and the tubular endosomal network induced by sorting nexing 12 (Snx12) are necessary for transporting TYLCV directly to the basal membrane of epithelial cells (Xia et al., 2018). These virus particles are then absorbed into the hemolymph via the filter chamber, penetrate into the primary salivary glands, and are ultimately discharged through salivary secretions (Czosnek et al., 2017). Consequently, the CP serves as the viral attachment protein that helps virions associate with various whitefly tissues. Studies of the *Tomato yellow leaf curl Sardinia virus* (TYLCSV) and AbMV suggest that CP amino acid residues 129 to 152 are crucial for crossing the gut/haemolymph barrier (Hohnle et al., 2001; Caciagli et al., 2009). Similarly, N-terminal region of CP in monopartite begomoviruses were found to be essential for penetration into the primary salivary glands (Wei et al., 2014). The interaction of whitefly egg vitellogenin with the 81–222 amino acid region of the CP is vital for transovarial transmission (Ghanim et al., 1998; Wei et al., 2017). A change in just one or two amino acid(s) in this region impairs insect transmission, thereby highlighting the importance of the N-terminal region of the CP in virus transport in insects (Noris et al., 1998).

The localization of TYLCV-CP in the microvilli of the gut lumen, gut epithelial cells, and midgut of the digestive tract, which implies a mode of virus translocation through insect gut cells (Czosnek et al., 2002). The CPs of various begomoviruses also interact with other gut proteins, such as collagen (Rana et al., 2019), cyclophilin B (Kanakala and Ghanim, 2016), midgut protein (Rana et al., 2016), and peptidoglycan recognition protein (Wang et al., 2016), to facilitate virus transmissibility. However, the interactions of the CP with HSPs serve various purposes in TYLCV transmission (Gorovits and Czosnek, 2017). In addition, the N-terminal domain of the CP binds to the GroEL chaperone of bacterial endosymbionts located in the whitefly midgut (Morin et al., 1999). These interactions may protect virions and assist in the crossing of whitefly hemolymph. However, excluding the organs involved in this circulative pathway, the penetration of viruses into other organs may be lethal to the insect vector. The enhanced expression of a whitefly gene known as knottin-1 (knot-1) has been detected upon TYLCV infection, and silencing of the knot-1 gene has been shown to increase the level of virus ingestion (Hariton-Shalev et al., 2016). From this study, the knot-1 protein appears to regulate the amount of virus particles that are taken up and transmitted by whiteflies.

CabLCV-BV1 and TYLCCNB-βC1 proteins participate in virus-vector interactions by binding to the MYC2 transcription factor and negatively regulate MYC2-dependent terpene synthase (TPS) genes (Li R. et al., 2014). Similarly, the TrAP of TYLCV binds competitively with ubiquitin, which results in the reduced ubiquitination of the JAZ1 (JASMONATE-ZIM DOMAIN1) protein and thereby prevents the MYC2-mediated induction of defense genes such as EAS (epi-arisotolchene synthase) and TPS (Li et al., 2019). The compromised regulation of terpene and other defense genes by these viral proteins helps increase whitefly performance in virus transmission (Figure 6B). In addition, proteomic analysis of diverse begomovirus-insect interactions suggests the probable contribution of several whitefly derived proteins in differential viral transmission (Zhao et al., 2019). Therefore, to establish mutuality with vectors, begomovirus proteins interact with various insect proteins and/or subvert plant resistance to whiteflies, which results in successful virus transmission from infected to healthy plants.

## Future Directions

Plant-infecting and insect-transmissible geminiviruses cause considerable yield losses in economically important crops worldwide. The spread of these diseases to newer hosts (crops and weeds) and previously non-endemic locations is increasing at an alarming rate, rendering them an emerging threat to global food security. This necessitates a collective effort to devise effective management approaches for diseases caused by these important plant pathogens. Because of their small genome, geminiviruses cannot afford to encode many proteins for the completion of their infection cycle. However, most of these multifunctional viral proteins can efficiently hijack several cellular machineries and function as pathogenicity determinants. To defend against geminiviruses, plants employ various strategies to create an unfavorable environment for virus propagation and restrict virus movement. Consequently, geminiviruses induce host genes and/or interact with plant proteins to suppress host-mediated defense responses, modulate the cell cycle, and even hijack host developmental and hormonal pathways. This enables the balanced co-evolution of plant and virus.

The recent experiments demonstrating novel antiviral methods employed by hosts are encouraging. However, many other new and potentially effective antiviral approaches need to be investigated. In addition, it is important to analyze the factors involved in virus infections between susceptible and resistant cultivars because the nature of a pathosystem is crucial to determining plant infection by particular viruses. While the general mechanisms of geminivirus movement have been elucidated, the macromolecular trafficking routes by which these viruses exploit plants at the cellular level are still poorly understood. Additional studies of such routes may provide insights into the identification of several unknown natural resistance factors and enable us to better understand host-virus interactions. Reports on geminiviral resistance factors and resistant cultivars are available. However, these viruses can overcome plant resistance through mixed infections or by replacing the ‘susceptible’ virus strains with mutated or recombinant ‘resistant’ strains. Such scenarios highlight the crucial need for in-depth analysis and characterization of the mutualistic interactions between geminiviruses and insect vectors, especially those that affect virus spread. A systems biology-based approach combining existing data (such as genome-wide transcriptomic, proteomic, and small RNA analyses between infected and healthy plants) with new data on susceptible and resistant cultivars should be taken to identify common plant target genes and/or proteins. In addition, it is important to integrate all direct and indirect plant–virus interaction networks that can help us design novel virus control strategies that do not impair plant growth and development.

## Author Contributions

RVK conceived and drafted the manuscript.

## Conflict of Interest Statement

The authors declare that the research was conducted in the absence of any commercial or financial relationships that could be construed as a potential conflict of interest.
